# Clinical and Biological Aspects of Disseminated Tumor Cells and Dormancy in Breast Cancer

**DOI:** 10.3389/fcell.2022.929893

**Published:** 2022-06-28

**Authors:** Alexander Ring, Maria Spataro, Andreas Wicki, Nicola Aceto

**Affiliations:** ^1^ Department of Biology, Institute for Molecular Health Sciences, ETH Zurich, Zurich, Switzerland; ^2^ Department of Medical Oncology and Hematology, University Hospital Zurich, University of Zurich, Zurich, Switzerland

**Keywords:** adjuvant therapy, chemotherapy, circulating tumor cells, disseminated tumor cells, metastasis, liquid biopsy, clinical trial design

## Abstract

Progress in detection and treatment have drastically improved survival for early breast cancer patients. However, distant recurrence causes high mortality and is typically considered incurable. Cancer dissemination occurs *via* circulating tumor cells (CTCs) and up to 75% of breast cancer patients could harbor micrometastatses at time of diagnosis, while metastatic recurrence often occurs years to decades after treatment. During clinical latency, disseminated tumor cells (DTCs) can enter a state of cell cycle arrest or dormancy at distant sites, and are likely shielded from immune detection and treatment. While this is a challenge, it can also be seen as an outstanding opportunity to target dormant DTCs on time, before their transformation into lethal macrometastatic lesions. Here, we review and discuss progress made in our understanding of DTC and dormancy biology in breast cancer. Strides in our mechanistic insights of these features has led to the identification of possible targeting strategies, yet, their integration into clinical trial design is still uncertain. Incorporating minimally invasive liquid biopsies and rationally designed adjuvant therapies, targeting both proliferating and dormant tumor cells, may help to address current challenges and improve precision cancer care.

## 1 Clinical Background

### 1.1 Breast Cancer Recurrence and Limitations of Adjuvant Systemic Therapy

Breast cancer is the most common non-cutaneous cancer in women worldwide (Ferlay et al., 2021). As a major achievement of surveillance efforts, most breast cancer cases (up to 93%) are initially diagnosed as localized or regionally advanced, potentially curable disease [Union for International Cancer Control (UICC) stage 1–3] (Noone et al., 2017). Surgery of the primary tumor (whenever possible) is the mainstay in therapy, frequently combined with neoadjuvant systemic therapy (i.e., before surgery) to reduce tumor burden, increase chances for complete resection and ideally achieve a complete eradication or pathologic complete response (pCR) (i.e., no detectable tumor at the time of surgery). The concept of pCR was endorsed by clinicians and major medical regulatory bodies as a surrogate for long term outcome of patients. However, a recent major meta-analysis including over 50 trials found no correlation between pCR and long-term outcome in breast cancer patients (Conforti et al., 2021).

The dearth of local response to predict outcome may be attributed to findings in preclinical models and patients that distant metastasis could occur in the early stages (Hüsemann et al., 2008a; Hosseini et al., 2016), with up to 75% of breast cancer patients harboring micrometastases at the time of diagnosis (Friberg and Nyström, 2015; Hu et al., 2019). Consequently*,* systemic adjuvant therapy (i.e., post-surgery) is aimed at eliminating residual or disseminated tumor cells (DTCs). Despite a reduction in mortality with the advent of adjuvant therapy, up to 41% of patients present with distant recurrence (i.e., metastatic breast cancer) up to 32 years after initial diagnosis and standard of care treatments (Tevaarwerk et al., 2013; Pedersen et al., 2022). While hormone receptor (HR)-positive disease can recur after decades, triple negative breast cancer (TNBC) patients surviving 5–8 years after surgery rarely relapse (Kennecke et al., 2010; Jatoi et al., 2011; Early Breast Cancer Trialists’ Collaborative Group, 2005) for reasons that are poorly understood.

Metastatic breast cancer remains largely incurable (Caswell-Jin et al., 2018), with strikingly lower 5-year survival in metastatic compared to non-metastatic patients (28% vs. 99%, respectively) (Noone et al., 2017), rendering metastasis the leading cause of breast cancer-related deaths in women (Gupta and Massagué, 2006). Shortcomings to achieve curative results may be attributed to several limitations of current clinical management strategies: 1) clonal composition and biology of disseminated cells and micrometastatic disease differs from larger tumor lesions. 2) DTCs enter a (initially indolent) state of dormancy that protects them from detection and eradication (Phan and Croucher, 2020), but eventually fuel distant relapse. 3) Current diagnostic tools (e.g., imaging, tumor biopsies) and systemic therapies are not designed to detect and target dormant disease.

In this review, we will discuss standard of care adjuvant treatment strategies and the use of biomarkers for more accurate patient stratification, including their limitations. The (re-) emerging concept and biology of cancer dormancy as a major impediment for long-lasting or definitive cure will be reviewed, with special emphasis on the role of circulating tumor cells (CTCs) in spreading dormant, yet lethal cancer. We will discuss novel approaches for the diagnosis and targeting of dormant cancer based on state-of-the-art knowledge and highlight future challenges and priorities.

### 1.2 Adjuvant Chemotherapy in Breast Cancer

Following a diagnosis of early stage breast cancer, the most immediate challenge is defining prognosis and an appropriate adjuvant systemic therapy regimen targeted at DTCs to achieve lasting cure. The beginnings of systemic chemotherapy and the idea of curing cancer can be traced back to the field of haemato-oncology and the revolutionary concept of combining several drugs in the form of polychemotherapy (Greenspan et al., 1963; DeVita and Chu, 2008). Adoption of Skippers ‘‘Cell Kill’’ hypothesis, which postulates that a given dose of drug kills a constant fraction of tumor cells rather than a constant number (Skipper et al., 1964), led to more aggressive use of chemotherapy and suggested that drugs effective against advanced (high-burden) disease might work equally or better in the adjuvant situation to eradicate micrometastases (Schabel, 1975). Adjuvant chemotherapy has evolved through various “generations” to achieve ever higher efficacy (Goldvaser et al., 2018). First generation regimens included treatment with CMF (cyclophosphamide, methotrexate and 5-fluorouracil), or anthracycline-based chemotherapy. The second generation introduced a higher number of cycles, higher doses of anthracyclines, the addition of paclitaxel to anthracyclines, and dose-dense regimens (Citron et al., 2003). Third generation treatments further escalated anthracycline and taxane-treatments, either concurrently or in sequence (Goldvaser et al., 2018). In early HR-negative cancer, cumulative improvement in 5-year disease-free survival (DFS) and overall survival (OS) ranged from 2.5% to 7.4% across different generations, while patients at high risk for relapse (tumors >2 cm, nodal involvement) and patients with HR-positive disease derived no (or smaller magnitude) in benefit (Goldvaser et al., 2018).

Provocatively stated, success of adjuvant chemotherapy over roughly 40 years came primarily through a strategy of treatment escalations. While successful in extending survival, this strategy potentially hampered further investigation of the underlying biology of micrometastatic disease, and a definitive cure for breast cancer patients requiring chemotherapy remains elusive.

The euphoria about the success of chemotherapy arguably also led to overtreatment of women either not in need of or not deriving benefit from adjuvant chemotherapy (Pak and Morrow, 2022; Ragusi et al., 2022). This is regrettable since toxicity is a major concern of past and current adjuvant strategies. Short-term toxicities (e.g., anemia, nausea, cytopenia and hepatotoxicity) can be threatening and therapy-limiting but are usually temporary and manageable. Long-term toxicities, including secondary cancers, heart failure, neurotoxicity, cognitive impairment and infertility, on the other hand, often last far beyond the treatment period with substantial impact on the quality of life of patients treated with curative intent (Burstein, 2020).

Consequently, the necessity of aggressive adjuvant therapy has been challenged (Kushner, 1984), due to increasingly only marginal benefit that requires treatment of large numbers of patients to derive relatively small improvements in outcome and limited to subgroups of patients (Goldvaser et al., 2018; Early Breast Cancer Trialists’ Collaborative, 1988), while causing considerable toxicity and financial burden (Burstein, 2020).

### 1.3 Adjuvant Drug Design—A Flawed Paradigm

Most standard of care and novel targeted cancer therapies, including immunotherapy, were developed based on preclinical studies measuring primary tumor shrinkage as the main indicator of efficacy (Anderson et al., 2019). Subsequent clinical studies in the palliative setting predominantly use RECIST (Response Evaluation Criteria In Solid Tumors) criteria, representing a set of published rules for tumor response that intentionally do not take into account clinical improvement or long-term outcome and relapse (Eisenhauer et al., 2009). Drugs for adjuvant treatment are usually “reutilized” after successful results in metastatic disease (often based on surrogate markers instead of overall survival, see below). In routine clinical practice and outside of clinical trials, these drugs are administered largely based on primary tumor biopsies in non-metastatic breast cancer. The limited success of this predominant treatment paradigm in the adjuvant-curative setting is well known and has recently been illuminated in a study by Parsons et al. (2020), which found that of 14 drugs recommended and used in the metastatic setting, only one (gefitinib in non-small cell lung cancer) was found to yield survival benefit for early-stage cancers.

The remarkable success of cyclin dependent kinase 4 and 6 inhibitors (CDK4/6i) in metastatic breast cancer (Goetz et al., 2017; Sledge et al., 2017) was put to the test in large prospective phase 3 trials in the adjuvant setting in early high-risk ER-positive/human epidermal growth factor receptor 2 (ERBB2/HER2)-negative disease, yielding contradictory results: while the monarchE trial reported a significant improvement of invasive disease free survival (IDFS) (Johnston et al., 2020), both the PALLAS and PENELOPE-B trial did not confirm these findings, showing no benefit (Loibl et al., 2021; Gnant et al., 2022). The tyrosine-kinase inhibitor lapatinib, which resulted in superior outcomes when added to cytotoxic chemotherapy in HER2-positive metastatic breast cancer (Geyer et al., 2006), did not improve outcomes when added to cytotoxic chemotherapy in the adjuvant setting (Piccart-Gebhart et al., 2016).

Further examples abound beyond breast cancer. In colorectal cancer, both irinotecan (a topoisomerase inhibitor) and bevacizumab (an anti-VEGF-A-antibody) have been successfully applied in the metastatic setting yet failed to show any significant benefits in the adjuvant setting in controlled randomized trials (Papadimitriou et al., 2011; Kerr et al., 2016). The anti-epidermal growth factor receptor (EGFR) monoclonal antibody cetuximab showed no benefit over placebo in the adjuvant setting (Alberts et al., 2012). In non-small cell lung cancer, targeted therapies with erlotinib and bevacizumab showed no efficacy in the treatment of early-stage disease (Kelly et al., 2015; Wakelee et al., 2017).

Currently, only 34.8% of drugs used in the metastatic setting are endorsed for adjuvant treatment for breast cancer, colorectal cancer or non-small cell lung cancer (Parsons et al., 2020). Partially explained by more rigorous criteria for drug approval and registration in early-stage disease, the observed lack in efficacy argues against a simple correlation and indicates fundamental difference in the biology of micrometastatic vs. macrometastatic disease, with the former not adequately targeted by reutilized drug application.

### 1.4 Unfavorable Effects of Anticancer Therapy

Beyond lack of efficacy, patient data and experimental models revealed that cytotoxic chemotherapy also has paradoxical effects on tumor relapse, promoting metastasis by enhancing vascular permeability and activation of inflammatory pathways, facilitating intra- and extravasation of cancer cells and promoting tumor dormancy (D'Alterio et al., 2020; Shiozawa et al., 2011; Brugger et al., 1994; Kurppa et al., 2020). A series of preclinical studies indicated that antiangiogenic therapies (e.g., targeting VEGFR) can enhance metastasis, potentially by the induction of hypoxic conditions (Ebos et al., 2009; Pàez-Ribes et al., 2009). Targeted therapies such as the BRAF inhibitor vemurafenib promote metastasis *via* AKT signaling (Obenauf et al., 2015). Low-dose cyclophosphamide has been shown to increase metastasis in lung adenocarcinoma or fibrosarcoma models (Man et al., 2008; Yamauchi et al., 2008). The same drug contributes to microenvironmental changes conducive to tumor cell survival, for example, by promoting the influx of pro-tumorigenic endothelial and monocyte progenitor cells (Hughes et al., 2015). Paclitaxel structurally resembles a pattern recognized by toll-like receptor-4 (TLR4) and can activate proinflammatory pathways (e.g., *via* macrophage activation) (Byrd-Leifer et al., 2001; Volk-Draper et al., 2014). Both cyclophosphamide and paclitaxel induce cancer cell dissemination *via* stress-response and Activating Transcription Factor 3 (ATF3) signaling (Chang et al., 2017). Chemotherapy can evict physiological cells from their niches and promotes tumor microenvironment (TME)-mediated cytokine release to recruit mesenchymal stem cells and establish a pre-metastatic niche as a sanctuary for DTCs (D’Alterio et al., 2020; Balkwill, 2004; Kaplan et al., 2006). Enhanced epithelial-mesenchymal plasticity *via* the microRNA (miR-21)/CDK5 axis and invadopodia formation in cancer cells triggered by chemotherapeutic drugs has been demonstrated in mouse models of breast cancer (Quintavalle et al., 2011; Ren et al., 2015). Direct evidence for pro-metastatic effects in humans and clinical scenarios is scarce and challenging to demonstrate conclusively. Nevertheless, indirect yet provocative data exists suggesting, for example, that patients receiving cyclophosphamide, taxanes, epirubicine, 5-fluoroacil exhibit suppressed expression levels of miR-488, an inhibitor of epithelial to mesenchymal transition (EMT) (Li et al., 2011) and that CTC numbers as a surrogate for metastatic spread increase post chemotherapy (Brugger et al., 1994). Together, these results highlight the need for a careful examination of risks versus benefits for standard cancer treatments as well as the need to identify the suitable response biomarkers.

### 1.5 Predictive Biomarker and Target Strategies in Breast Cancer

Significant efforts have been made to design rational systemic therapies to maximize efficacy and minimize side effects. The standardized assessment of estrogen receptor (ER)- (75% of breast cancer patients) and HER2-expression (25% of breast cancer patients) as predictive biomarkers enabled targeted therapy tailored to molecular breast cancer subtypes (Allison et al., 2020). Adjuvant antihormonal therapy in ER-positive breast cancer up to 10 years yielded profound survival benefits (Davies et al., 2013), but relapse, including beyond 10 years, still occurs in up to 17% of patients (Pedersen et al., 2022). Multiparametric gene expression profiles [e.g., MammaPrint, Oncotype DX, Endopredict, and Prosigna (PAM50)] have been designed with considerable success to further stratify early-stage HR-positive patients (i.e., ER-positive or progesterone receptor/PR) according to relapse risk (Kwa et al., 2017). Both Oncotype DX (Sparano et al., 2018; Kalinsky et al., 2021) and MammaPrint (Piccart et al., 2021) have been validated in prospective trials, confirming their predictive power for a subset of (high risk) breast cancer patients regarding duration of adjuvant anti-hormonal therapy or benefit of additional chemotherapy. On the downside, Oncotype DX was not predictive of benefit from chemotherapy escalation in HR-positive high-risk patients (Mamounas et al., 2018). The OPTIMA trial will prospectively further validate gene-expression-directed chemotherapy decision in high-risk patients (ISRCTN42400492). Two noteworthy biomarkers are uPA (urokinase-type plasminogen activator) and PAI-1 (plasminogen activator inhibitor-1), which showed promising results regarding prognostic and predictive validity for adjuvant chemotherapy in lymph node-negative HR-positive early breast cancer but are not widely used in the clinic (Harbeck et al., 2013).

Anti-HER2 targeted antibodies (e.g., trastuzumab, pertuzumab), concurrently or sequentially added to chemotherapy, substantially decreased recurrence in early-stage HER2-positive patients (Romond et al., 2005; Joensuu et al., 2006). This success led to further trials with the addition of kinase inhibitors but yielded disappointing results (Piccart-Gebhart et al., 2016; von Minckwitz et al., 2017; Chan et al., 2016). Remarkably, one such kinase inhibitor (neratinib) received Food and Drug Administration (FDA) approval despite its underwhelming efficacy, but also raised questions about the need for further, more accurate patient stratification (Lambertini et al., 2017; Miller, 2017), for example, using activating HER2-mutations in patients with normal or slightly elevated expression levels (Yi et al., 2020).

In the setting of immunotherapy programmed cell death protein 1 (PD-1)/programmed cell death 1 ligand 1 (PD-L1), tumor mutational burden (TMB) and tumor-infiltrating lymphocytes (TILs) are dependable (though not ideal) biomarkers in various tumor entities to predict the success of immune checkpoint inhibition, but potential value in breast cancer is so far limited to early TNBC and data are still regarded as immature (Teng et al., 2015; Goodman et al., 2017).

Very recently, germline mutational status of the DNA-repair genes breast cancer 1 and 2 (gBRCA1/2) has been shown to be predictive of response to the poly(ADP-ribose)-polymerase 1 (PARP1)-inhibitor olaparib in early-stage HER2-negative breast cancer patients (Tutt et al., 2021). Several additional predictive biomarkers have been clinically validated in the metastatic setting, such as estrogen receptor 1 (ESR1) mutational status predicting endocrine resistance (Turner et al., 2020a), phosphatidylinositol-4,5-bisphosphate 3-kinase catalytic subunit alpha (PIK3CA) (André et al., 2019) or neurotrophic receptor tyrosine kinase (NTRK) fusions (Hong et al., 2020), but have (so far) no application in the adjuvant setting.

Stratifying patients to predict efficacy of non-targeted chemotherapy proved even more difficult. Mathematical modeling (Norton-Simmons hypothesis) has been applied to identify more effective and less toxic treatment regimens (Simon and Norton, 2006). The CREATE-X trial demonstrated that breast cancer-subtype (TNBC) can predict benefit from escalated adjuvant chemotherapy (Masuda et al., 2017).

Besides the need for adequate mechanistical and clinical studies for predictive biomarker development and application, their use in clinical practice can be limited due to discrepancies between medical associations [e.g., European Society for Medical Oncology (ESMO) and National Comprehensive Cancer Network (NCCN)] (Zagouri et al., 2015), resulting in differences regarding approval by regulatory bodies [i.e., FDA and European Medicines Agency (EMA)].

## 2 Micrometastatic Disease and Dormancy in Breast Cancer

The discrepancy in efficacy comparing the success of current systemic therapies in reducing overt tumor burden on one hand and preventing relapse on the other maybe akin to a numbers game. Primary tumors and macrometastatic lesions consist of billions of polyclonal, often highly proliferative cells, dramatically increasing the likelihood of drugs to “find” fractions of sensitive cells. Eventually though, resistant clones will be selected, limiting efficacy (Aceto et al., 2014). Micrometastasis, on the other hand, represents rare DTCs in the form of single cells or oligoclonal cell clusters that survive dissemination and homing and likely possess protective biological properties, including the capacity to enter dormancy. Hence, consideration of the molecular characteristics of dormant DTCs represents an outstanding opportunity to improve the clinical management of early breast cancer (Braun et al., 2000).

### 2.1 Of Circulating and Disseminated Tumor Cells

**FIGURE 1:**
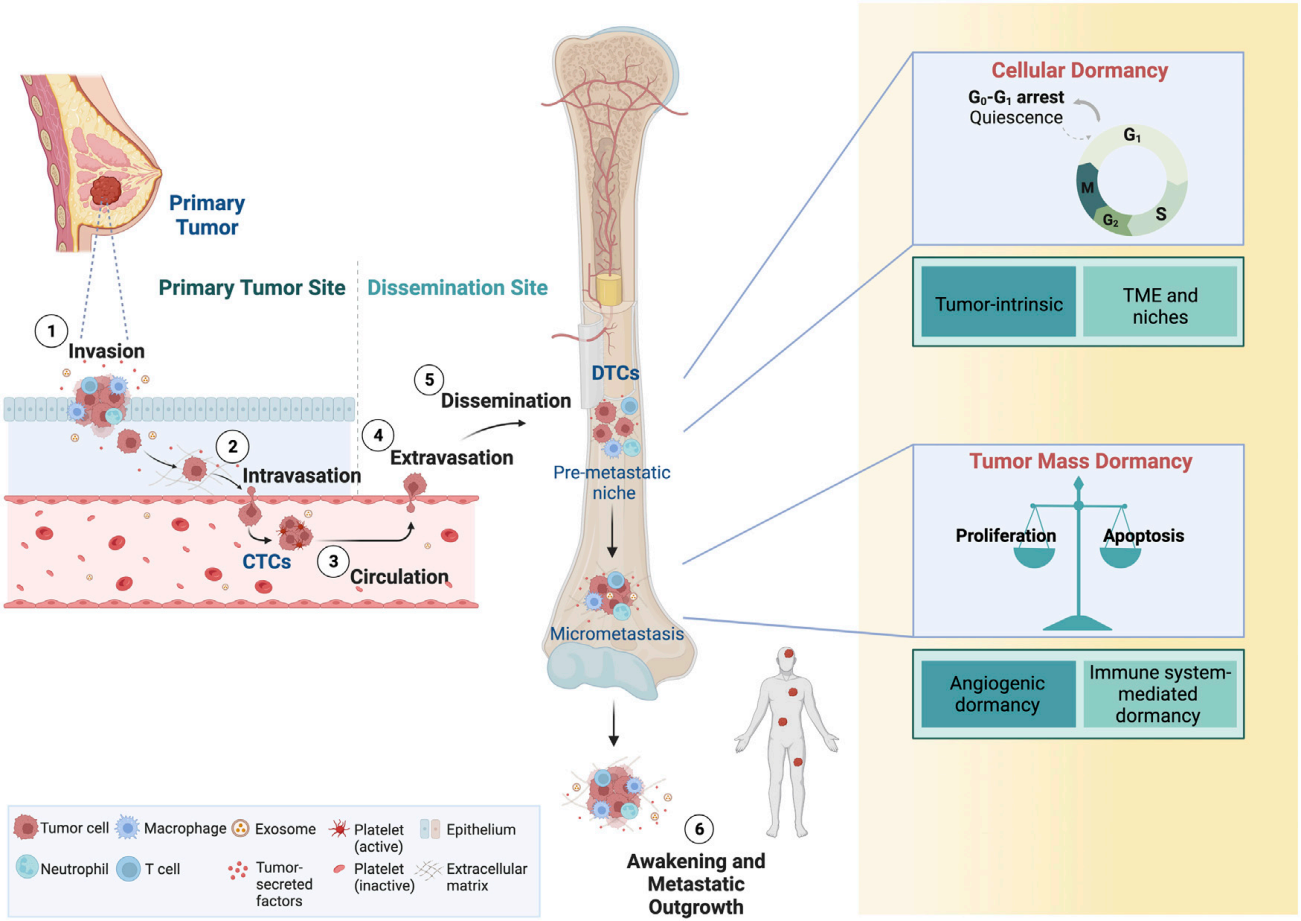
Dissemination of CTCs and tumor dormancy. Breast cancer cells are shed in the proximal circulation where they are referred to as CTCs. Single CTCs or clusters of CTCs can disseminate and home to distant organs sites, where they are referred to as DTCs. DTCs are found predominantly in the bone marrow, but also lung, liver and the brain. While cellular dormancy typically refers to single DTCs or clusters of DTCs, dormancy of micrometastases also occurs, and these are characterized by a transient cell cycle arrest or an equilibrium of proliferation/apoptosis, respectively. DTCs can reawaken, eventually giving rise to macrometastatic lesions. CTCs, circulating tumor cells; DTCs, disseminated tumor cells. Adapted from “Overview of Metastatic Cascade,” by BioRender.com (2022). Retrieved from https://app.biorender.com/biorender-templates.

Breast cancer cells that leave the primary tumor *via* the proximal circulation are tumor-derived pioneers of the metastatic process (Figure 1). Shedding of these circulating tumor cells (CTCs) occurs as single cells or homotypic and heterotypic CTC clusters (Aceto et al., 2014; Szczerba et al., 2019). Heterotypic CTC clusters are formed with several cell types, including white blood cell. Utilizing patient samples and models, it has been shown that clusters of CTCs occur in early and late stages of breast cancer, which possess superior capabilities to survive in circulation and in seeding metastatic lesions (Aceto et al., 2014; Gkountela et al., 2019; Donato et al., 2020; Krol et al., 2021). The successful propagation of CTCs involves multiple cell-intrinsic qualities and extrinsic cues from the tumor microenvironment (TME) and the immune system (Sznurkowska and Aceto, 2021). Eventually, CTCs and their clusters extravasate from circulation and spread to organs such as the bone marrow (BM), where they are referred to as DTCs. Intriguingly, not only CTCs but also DTCs may occur as clusters in breast cancer patients (Woelfle et al., 2005).

**FIGURE 2:**
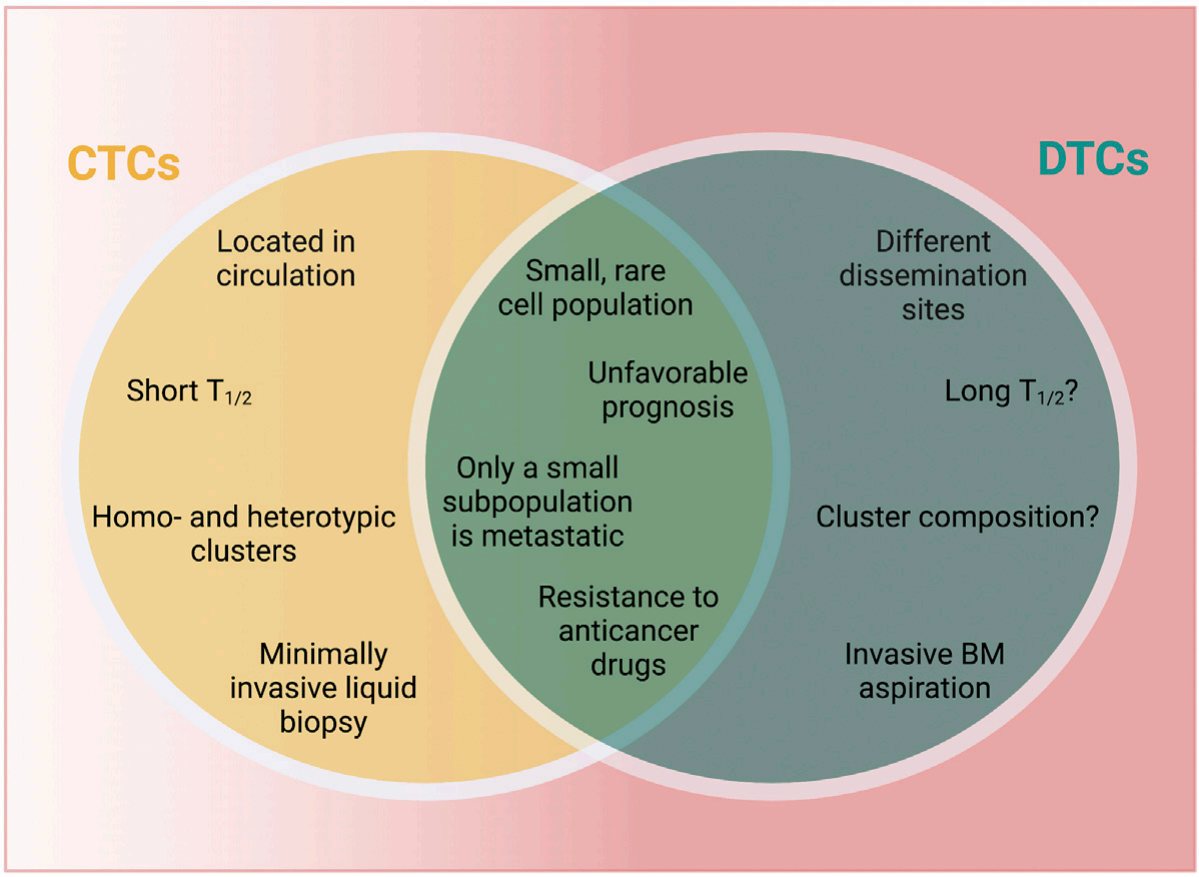
CTCs vs. DTCs. CTCs and DTCs are characterized by both unique and shared characteristics. Aspects that are unknown at this point are marked by “?” T_1/2_ = half-life. BM, bone marrow; CTCs, circulating tumor cells; DTCs, disseminated tumor cells. Created with https://biorender.com.

CTCs and DTCs share similarities in phenotypic plasticity and adaptability, which appear to be a hallmark of metastasis-competent cells (Yuan et al., 2019). Using mouse models and breast cancer patients, it has been shown that hypoxic CTC clusters have higher metastatic potential, while other preclinical studies showed that hypoxia can induce a dormant state in cancer (Fluegen et al., 2017; Donato et al., 2020), and that those hypoxic cells drive recurrence (Harada et al., 2012). It is intriguing to speculate that the propensity for dormancy is present as a cancer cell-intrinsic property and propagated *via* CTCs to distant sites. Supporting this notion are findings of dormant CTCs in the circulation of breast cancer patients (Spiliotaki et al., 2014; Vishnoi et al., 2015). Further similarities between CTCs and DTCs point towards an intricate connection of both cell types as part of the continuum of cancer metastasis: 1) both are rare cell populations and 2) their detection in blood or BM, respectively, is associated with an unfavorable prognosis. 3) not all CTCs or DTCs form metastasis but 4) have been associated with drug resistance (Figure 2). CTCs and DTCs are exceedingly rare in the blood stream [one CTC per milliliter blood (Paterlini-Brechot and Benali, 2007; Alix-Panabières and Pantel, 2014)] or BM (solitary DTCs or DTC clusters composed of 10–20 individual DTCs) of patients (Riethdorf and Pantel, 2008; Risson et al., 2020). The detection of these rare cell populations among oodles of other cells resembles the proverbial search for the needle in the hay (Riethdorf and Pantel, 2008; Risson et al., 2020), rendering methods to detect and isolate CTCs (Ramos-Medina et al., 2021) and DTCs challenging, yet highly sought after (Effenberger et al., 2011; Park, 2011).

Both CTCs and DTCs can be detected in a fraction of breast cancer patients when considering limiting sampling opportunities (e.g., few milliliters of peripheral blood), highlighting their importance in the metastatic process. Despite their relatively small numbers, CTCs and DTCs are prognostic for progression free survival and overall survival in breast cancer, largely independent of other factors as well as one another (Cristofanilli et al., 2004; Braun et al., 2005). The prognostic impact likely relates to their capability to mediate metastasis, although not all patients positive for CTCs and/or DTCs eventually present with overt metastasis (Wiedswang et al., 2006; Klein, 2009; Baccelli et al., 2013). CTCs and DTCs may also participate in primary tumor relapse and tumor self-seeding has been described (Kim et al., 2009). The identity of metastasis-competent subpopulations is not fully resolved and subject of intense research effort. While adjuvant therapy in principle is intended to eradicate CTCs and DTCs, metastatic relapse is common and hence, targeting strategies are so far incompletely realized (Naumov et al., 2003; Pavese and Bergan, 2014). While approaches to target DTCs will be examined in more detail below, a comprehensive evaluation of CTC targeting strategies is beyond the focus of this review. Worthwhile mentioning here is the exciting finding that pharmacological dissociation of CTC clusters can mitigate metastasis in mouse models (Gkountela et al., 2019), which is currently being validated in an early phase 1 clinical trial (NCT03928210).

It is important to mention that CTCs and DTCs are also characterized by relevant differences, including 1) localization and accessibility, 2) half-life, 3) frequency of detection and 4) cellular composition (Figure 2). CTCs are found in the blood stream, while DTCs lodge in various organs beyond BM (e.g., lymph nodes, lung, liver or brain) as demonstrated in preclinical (models) (Pantel and Brakenhoff, 2004; Sosa et al., 2011; Correia et al., 2021; Riggio et al., 2021; Tallón de Lara et al., 2021; Dai et al., 2022) and patients, including biopsy samples and autopsy studies (Noltenius and Noltenius, 1985; Kubuschok et al., 1999; Schimanski et al., 2003; Conzelmann et al., 2005; Sproll et al., 2017). The unfavorable conditions in circulation (e.g., shear stress-mediated effects, anoikis or immune cell aggression) greatly limit CTCs survival and circulation time (Aceto et al., 2014; Micalizzi et al., 2017). While DTCs may persist for decades (Holmgren et al., 1995), little is known about their clearance rate and their actual half-life, which would provide important insights into timing of metastatic progression. Differences in localization and half-life also affect ease or rate of detection. One prospective trial in breast cancer patients suggested that CTCs were less frequently detected than DTCs, however, whether this difference is due to the sensitivity of the detection methods or based on biological conditions remains unknown (Wiedswang et al., 2006). CTCs are detected from a liquid biopsy, which can be performed in a minimally invasive fashion, while DTCs have to be isolated by invasive means, usually *via* a BM aspirate (Wiedswang et al., 2006). Finally, while cell-cell-junctions in CTCs can lead to the formation of highly metastatic homotypic and heterotypic clusters observed in patients and *in vivo* models (Aceto et al., 2014; Liu et al., 2019; Szczerba et al., 2019; Donato et al., 2020; Taftaf et al., 2021) little is known about the role of multicellular aggregates in the DTCs context. Elucidating the composition and biological features of DTC clusters would likely further our understanding of breast cancer dormancy, including the discovery of novel biomarkers and innovative therapeutic approaches.

### 2.2 The Fate of Circulating Tumor Cells/Disseminated Tumor Cells and Clinical Evidence for Dormancy

Two mechanistic models of cancer cell dissemination have been proposed, namely the linear (also referred to as late dissemination) and the parallel progression model (also referred to as early dissemination) (Klein, 2009). The linear model relies on the principle of a tumor cell’s capability to undergo natural selection within the primary tumor, eventually leading to dissemination of the fittest (dominant) polyclonal tumor cells that will give rise to metastasis in distant organs (Kerbel et al., 1990; Klein, 2009). However, comparative analysis revealed in some cases genetic discrepancies between DTCs and primary tumors (Schmidt-Kittler et al., 2003; Aguirre-Ghiso, 2007). The parallel progression model postulates that dissemination of tumor cells occurs at early stages, and occasionally, even before the primary tumor becomes clinically manifested (Klein, 2009; Hu et al., 2020). This has been accepted among a portion of the research community, however, the linear progression model is not to be excluded. It is conceivable to assume that timing of dissemination is contingent upon a variety of factors, and that a spectrum of various situations ranging from early to late exists when considering large groups of patients. Since the timing of tumor cell dissemination and features that lead to the outgrowth of disseminated cells are still not fully elucidated (Hüsemann et al., 2008b), the field of dormancy would greatly benefit from accurate cancer models and additional clinical data.

Following extravasation from the blood stream, CTCs are incisively subjected to distinctive environments, resulting in divergent fates with fundamental impact on patient outcomes. The likely predominant outcome is the killing of large numbers of tumor cells because of unfavorable conditions (e.g., immune surveillance), therefore posing limited risk for the patient. On the opposite end of this spectrum of possible outcomes, disseminated cells might undergo rapid cell divisions resulting in life threatening metastatic lesions. As a third possibility, DTCs might survive their initial transit but subsequently enter a nonproliferative state termed dormancy, which is the basis of clinical latency and eventual overt metastatic relapse (Aguirre-Ghiso, 2007). Dormancy can occur at the cell intrinsic level (i.e., cellular dormancy) or at the level of micrometastases (i.e., tumor mass dormancy) (Aguirre-Ghiso, 2007). Cellular dormancy is characterized by single, non-proliferating tumor cells entering temporary and reversible cell cycle arrest, also known as quiescence (Aguirre-Ghiso, 2007; Riethdorf and Pantel, 2008; Phan and Croucher, 2020). Key features of cellular dormancy are resistance against antiproliferative drugs, escape from immune system-mediated clearance and dynamic entry and exit from the quiescent state (Phan and Croucher, 2020). In contrast, tumor mass dormancy occurs when cell proliferation and cell death are kept in balance, usually *via* apoptosis, preventing tumor mass expansion (Phan and Croucher, 2020). This steady-state condition can be driven by various factors, including a lack of angiogenesis or by immune system-mediated factors (Aguirre-Ghiso, 2007). Microscopic tumor cell clusters that are devoid of vasculatures can persist imperceptibly for month or even years in a clinically disease-free patient and only give rise to metastasis upon acquiring angiogenic characteristic promoting neovascularization (Naumov et al., 2006). Both cellular and tumor mass dormancy can coexist before the detection of overt (metastasis) (Phan and Croucher, 2020).

Although the concept of dormancy has been called into question (Uhr and Pantel, 2011), both clinical evidence as well as experimental and mathematical models support findings that clinical latency is not simple the result of slowly proliferating tumor cell. Patients presenting with BM resident DTC populations that exhibit decreased expression of proliferation markers [e.g., marker of proliferation Ki-67, proliferating cell nuclear antigen (PCNA)], as well as the presence of CTCs up to 20+ years after initial treatment without overt tumor lesions argues for a reservoir of dormant cancer cells that can shed into circulation (Sauer et al., 2021). Metastatic cancers of unknow primary and cancer after organ transplant from seemingly cancer-free donors further argue for the existence of dormant DTCs (Sauer et al., 2021). The commonly used Gompertzian function to predict tumor size and growth based on clinical data analysis presumes an initial exponential growth phase followed by a decrease in proliferation, without accounting for dormancy, resulting in discrepancies in expected and actual tumor size (Page, 2009). Several mathematical concepts have been applied to further our understanding of driving processes in tumor dormancy [reviewed in (Page, 2009)].

The exploration of features that cause tumor dormancy have spawned and dominated an entire research field over the past years, resulting in a plethora of cues which altogether can be categorized in cell intrinsic or extrinsic factors that promote dormancy and allow escape from physiological and therapeutic elimination strategies of DTCs.

### 2.3 Dormancy Factors

#### 2.3.1 Cell-Intrinsic Factors

A major driver of dormancy is the balance of mitogen-activated protein kinase (MAPK) p38 and extracellular signal-regulated kinase (ERK1/2) activity. While p38 activation results in the inhibition of cyclin D1 transcription and cell cycle arrest, ERK activation exerts the opposite effect *via* the stimulation of cyclin D1 transcription and cell cycle progression (Lavoie et al., 1996). Hence, cellular dormancy is typically associated with low levels of ERK and high levels p38. The G0-G1 cell cycle arrest caused by the ERK^low^/p38^high^ can also be regulated through cyclin D1-independent mechanisms such as activation of p53, upregulation of nuclear receptor subfamily two group F member 1 (NR2F1) and basic helix-loop-helix domain containing, class B3 (BHLHB3) or downregulation of forkhead box M1 (FOXM1) and jun proto-oncogene (c-Jun) (Adam et al., 2009; Sosa et al., 2011; Sosa et al., 2015). The Dual Specificity Tyrosine Phosphorylation Regulated Kinase 1A (DYRK1A) causes prolongation of the G1 cell cycle phase and cell cycle arrest *in vitro*, which also occurs in a cyclin D1-dependent manner (Chen et al., 2013). Johnson et al. (2016) demonstrated *in vivo* and *in silico* the contribution of leukemic inhibit factor receptor (LIFR) and signal transducer activator 3 (STAT3) to stimulate breast cancer dormancy. Loss of LIFR and STAT3/Suppressor Of Cytokine Signaling 3 (SOCS3) resulted in reactivation of dormant tumor cells and bone colonization. These findings were corroborated by an analysis of the The Cancer Genome Atlas (TCGA), showing that breast cancer patients who had developed bone metastasis, compared to those not affected by dissemination to the bone, had significantly reduced gene expression of LIFR and STAT3 (Johnson et al., 2016). Another retrospective analysis of breast cancer patient genomic data suggested that other genomic alterations (e.g., CNAs) are correlated with late relapse (Rueda et al., 2019). Expression of stem cell-related transcription factors, including SOX2 and SOX9, enforce dormancy in breast cancer DTCs in pre-clinical models (Malladi et al., 2016) and patient-derived xenograft (PDX) models indicated that stem cell-like characteristics and epithelial-mesenchymal plasticity contribute to a dormant phenotype in metastatic tumor cells (Lawson et al., 2015a). Single-cell RNA sequencing data from pre-clinical models implicated metabolic-hemostatic changes (i.e., increased mitochondrial respiration) in successful metastatic seeding (Davis et al., 2020).

Ultimately, dormancy often results from “funneling” heterogenous molecular circuits towards the induction of cell cycle inhibitors, in particular cyclin dependent kinase inhibitors (CDKN) 1A, CDKN1B or CDKN2A as shown in preclinical models (Adam et al., 2009; Demaria et al., 2017; Fukushima et al., 2019).

#### 2.3.2 Angiogenic Factors, Tumor Microenvironment and Niches

In multiple organs (e.g., lung, bone, brain or liver) DTCs reside in the perivascular niche and angiogenic factors have been propelled to the forefront of investigations into tumor dormancy (Ghajar et al., 2013; Correia et al., 2021). A methodical investigation of the BM vasculature in mice by Carlson et al. (2019) using the 4T07 syngeneic TNBC model showed that DTCs located in the perivascular niche were protected against antiproliferative drug effects *via* the dormancy promoting influence of the vascular endothelium. Additional experiments revealed the role of integrin isoforms B_1_ and/or a_v_b_3_ in inducing a dormant phenotype, and anti-integrin targeted therapy caused a switch to a chemo-sensitive phenotype and killing of DTCs (Carlson et al., 2019). Several other antiangiogenic proteins have been demonstrated to promote dormancy in preclinical models, for instance thrombospondin-1 (Ghajar et al., 2013), angiostatin (O’Reilly et al., 1996), transforming growth factor *β* (TGF-β) (Ghajar et al., 2013) and phosphoinositide 3-kinase (PI3K) (Shor et al., 2022). Hypoxia has been shown to be a potent inducer of tumor dormancy, for example, *via* the upregulation of the transcription factor NR2F1 and of the dormancy genes CDKN1B and DEC2 (also known as basic helix-loop-helix family member E41) in patient-derived and transgenic mouse models (Fluegen et al., 2017). These findings notwithstanding, a recent study (in xenograft models and patients) proffered hypoxic CTC cluster to be characterized by higher metastatic potential compared to normoxic clusters (Donato et al., 2020), demonstrating the need for a more granular understanding of the role of hypoxia in dormancy and metastasis.

The TME has been shown to be crucial for tumor dormancy initiation and maintenance in breast cancer cells (Bakhshandeh et al., 2022) *via* paracrine/niche-related signaling [e.g., bone morphogenetic protein (BMP) 4 and 7 (Kobayashi et al., 2011; Gao et al., 2012), TGF-β2 (Bragado et al., 2013) and type 3 collagen (Di Martino et al., 2022)] that can maintain cancer cell dormancy in experimental models. In the BM as the most studied sanctuary for DTCs, three supporting niches have been described. Beside the above-described perivascular niche, attention has recently been brought to the endosteal niche (also referred to as osteoblastic niche) (Lawson et al., 2015b; Croucher et al., 2016). On the endosteal surface, disseminated breast cancer are protected from the effects of antiproliferative drugs *via* dormancy induction by bone-forming cells, or osteoblasts. Conversely, a remodeling of the osteogenic niche by bone-degrading cells, or osteoclasts, causes cell cycle reentry of DTCs and the formation of macrometastases in mice *via* osteoclast-mediated transfer of calcium from bone to DTCs and activation of calcium-associated signaling (Lawson et al., 2015b; Wang et al., 2018). Survival of DTCs lodged in the BM also seems dependent on counteracting tumor Necrosis Factor Related Apoptosis Inducing Ligand (TRAIL)-mediated clearance *via* SCR signaling and C-X-C Motif Chemokine Receptor 4 (CXCR4)—C-X-C Motif Chemokine Ligand 12 (CXCL12) signaling by co-opting the hematopoietic stem cell niche (Zhang et al., 2009).

Other organs can harbor dormant breast cancer DTCs but have been less well characterized. Notable exceptions are recent findings that hepatic stellate cells can counter natural killer cell (NK) cell-mediated cancer dormancy in syngeneic breast cancer mouse models (Correia et al., 2021) and that astrocyte-deposited laminin-211 drives quiescence of DTCs homing to the brain of TNBC mouse models (Dai et al., 2022).

#### 2.3.3 Immune System-Mediated Dormancy

Immune system-mediated therapy resistance of tumor cells is of paramount importance in the cancer dormancy field (Teng et al., 2008). An elegant explanation for immune evasion of micrometastatic tumors was offered by the “3 Es” of the so-called immunoediting concept described by Dunn et al. (2002) (Teng et al., 2008). Failure of the immune system to kill aberrant cells (during the *Elimination* phase) results in an *Equilibrium* state, or dormant state, during which proliferation and tumor-suppressive immune response are in a steady-state condition (Vesely et al., 2011). During this phase, cancer cells acquire mutations that lead to a competitive advantage. *Escape*, which ultimately leads to an increased proliferation and unchecked tumor growth, leads to the formation of macrometastases (Vesely et al., 2011). Whether and how this concept relates to quiescent cancer cells remains to be explored. DTCs and micrometastases downregulate or entirely lose major histocompatibility complex I (MHC I) expression, thereby evading clearance by the adaptive [i.e., cluster of differentiation (CD) 4^+^ and CD8^+^ positive T cells] and innate (e.g., NK cells) immune system. This was explored in a clinical trial collecting BM aspirates from breast cancer and other cancer entities, demonstrating the most pronounced reduction of MHC I expression in breast cancer (Pantel et al., 1991). Accordingly, loss of MHC I correlates with worse prognosis in TNBC as shown by proteomic profiling of the human disease in primary tumors (Pedersen et al., 2017). CD4^+^ and CD8^+^ further control dormant tumor cells through secretion of interferon *γ* as demonstrated in mouse models (Koebel et al., 2007). DTCs can escape NK cell-mediated elimination through the expression of the wingless-related integration site (WNT) antagonist dickkopf WNT signaling pathway inhibitor 1 (DKK1), as demonstrated in lung adenocarcinoma and HER2-positive breast cancer mouse models (Malladi et al., 2016). Cytoskeletal changes, such as the accumulation of filamentous actin (F-actin) at immune synapses between tumor and immune cells, and autophagy of granzyme B have been linked to increased resistance to NK cell lysis as indicated in *in vitro* and *in vivo* experiments (Baginska et al., 2013; Al Absi et al., 2018).

Although significant strides have been made towards the identification of key players in dormancy, this knowledge has yet to be translated into improved clinical management and outcomes in cancer patients. Currently, no reliable diagnostic or targeted therapeutic tools exist to selectively tackle cancer dormancy and its lethal outgrowths.

## 3 Opportunities Ahead

### 3.1 Limitations of Current Medical Imaging and Biopsy Approaches

Imaging is a mainstay in breast cancer surveillance and diagnosis and mammography is the most widely used imaging modality for initial diagnosis. While considered highly accurate with high positive (89%) and negative predictive values (91%), mammograms have been regarded extremely controversially due to high risk of false positive results and overdiagnosis, with estimates ranging from 1 in 3 (Bleyer and Welch, 2012) to more recently 1 in 7 overdiagnosis (Ryser et al., 2022), leading to overtreatment and potential harm that outweighs benefit (Pak and Morrow, 2022; Ragusi et al., 2022). These results notwithstanding, a recent study estimated that detecting 50% of distantly spread breast cancer (i.e., late-stage) cases in a local or regional (i.e., early) stage would lead to a reduction in cancer-specific mortality of 21% over a 10-year period (Yu et al., 2022). Hence, early detection, including dormant macrometastatic disease, is paramount to improve outcomes. Micrometastasis or single DTCs are far too small for the detection limit of current imaging modalities. Furthermore, medical imaging does not have the capability to determine molecular or predictive features. Novel developments (e.g., radiomics applying data-characterization algorithms to medical imaging data, or photoacoustic imaging) (Fotopoulou et al., 2021; Lin and Wang, 2022) are being investigated, but these technologies likely will not be evenly distributed and implemented or validated for widespread use in clinical trial designs anytime soon (Weissleder et al., 2016).

Tissue samples are the gold standard for deriving predictive information but have several limitations: 1) sampling bias due to intra- and intertumoral heterogeneity, 2) inaccessibility, including multiple metastatic sites in difficult-to-access anatomical locations, 3) impracticality, including medical contraindications and patients’ hesitation to undergo biopsy due to their invasive, painful and at times risky character. Consequently, one third of patients with metastatic cancer in prospective clinical trials do not have biopsy results available (André et al., 2014).

### 3.2 Improving Diagnosis: The Clinical-Translational Potential of Liquid Biopsies

Liquid biopsies are usually performed on blood *via* peripheral venipuncture but can be performed on all body fluids. Compared to tissue biopsies they are minimally invasive, can be collected frequently and repeatedly, usually have faster turnaround times, dramatically improve early detection of minimal residual disease (MRD) or molecular relapse (MR) and potentially represent cancer heterogeneity more faithfully [comprehensively reviewed by Ignatiadis et al. (2021)].

In extension, “liquids” may be used for early detecting and targeting of dormant residual or micrometastatic disease in patients at high risk of relapse (Barrière et al., 2012; Shaw et al., 2012; Bettegowda et al., 2014; McDonald et al., 2019; Thery et al., 2019; Krol et al., 2021), which may prevent early cancers to evolve into difficult-to-treat macrometastatic tumor lesions. Though these technologies still need to be benchmarked against current diagnostics tools to establish their role in clinical practice (i.e., superiority or complementarity) using adequate clinical trial design, many promising developments are currently under way.

#### 3.2.1 Circulating Tumor Cells as Biomarkers in Disseminated Cancers

CTCs are pioneers of the metastatic process and present in all stages of breast cancer (Cristofanilli et al., 2004; Xenidis et al., 2009; Bidard et al., 2010), sometimes many years after removal of the primary tumor or without overt disease (Meng et al., 2004) and even at the stage of “pre-malignant” lesions such as DCIS, where 50% fatalities occur *via* metastasis (after surgery, without local recurrence) (Banys et al., 2012; Narod et al., 2015). In breast cancer patients, the presence of CTCs correlates with the presence of DTCs (Fehm et al., 2009). It has been shown that the risk of late recurrence is the same for small and large breast primary tumors and DTC counts do not correlate with tumor size (Demicheli et al., 1996; Braun et al., 2005). Taken together, the findings indicate that cancer cell dissemination *via* CTCs might occur at any timepoint (Schmidt-Kittler et al., 2003; Schardt et al., 2005), and implicates the seeding of dormant micrometastatic lesions.

Cell surface marker or epitope-dependent and -independent technologies are available for CTC isolation (Descamps et al., 2022). While the first FDA approved device (CellSearch) is epitope-dependent (i.e., epithelial cell adhesion molecule (EpCAM) and cytokeratin expression on CTCs), marker-independent isolation and enrichment devices have been recently gaining popularity and acquired CE Mark regulatory approval for the clinical market in Europe as well as FDA approval in metastatic breast cancer (i.e., Angle Parsortix) (Miller et al., 2018). Though not currently implemented in clinical practice, CTCs are part of the UICC classification of malignant tumors (TMN) and have demonstrated prognostic potential in numerous trials (Schochter et al., 2019). Less is known about the validity of CTCs as predictive biomarkers, although their potential utility for therapeutic decision making has been demonstrated, for example, in the PROPHECY trial using the presence of androgen receptor (AR)-splice variant 7 (V7) in CTCs (mRNA or protein) as an independent predictor of unfavorable progression-free survival and overall survival with anti-androgen therapy (Armstrong et al., 2019).

CTCs have an important advantage over other liquid biopsy analytes because they may represent surviving clones capable to escape and spread, hence can be considered most relevant to cancer progression. As intact cells, CTCs contain all components for multi-omics analyses and are amenable to functional studies (e.g., culturing, xenograft and avatar models, drug phenotyping). It was recently demonstrated that simultaneous assessment *via* whole exome sequencing (WES) and matched single-cell transcriptomics as well as drug phenotyping can be achieved from individually purified breast cancer CTCs from patients and xenograft models (Szczerba et al., 2019). Capturing tumor (temporal and spatial) heterogeneity by single cell CTC profiling (Keller and Pantel, 2019) is feasible and may enable the detection of dormancy precursors as well as predictive biomarker for targeting dormant disease. Early diagnosis of cancer *via* CTCs is a matter of debate and under active investigation with promising results in early-stage breast cancer, hinting at the possibility to detect small lesions, potentially including micrometastases and MRD (Barrière et al., 2012; Thery et al., 2019; Krol et al., 2021).

#### 3.2.2 The Role of Circulating Tumor DNA

Circulating tumor DNA (ctDNA) represents a fraction of the pool of circulating cell-free DNA and relies on the detection of tumor-specific genomic or epigenetic aberration compared to the germline background. When collected appropriately ctDNA samples are stable, facilitating centralized testing for multicenter trials and routine clinical use (Hrebien et al., 2016; Kang et al., 2016).

Several mutation-based detection assays have been developed and FDA approved (e.g., PI3K in breast cancer, EGFR in non-small cell lung cancer) that exhibit excellent specificity (mean 96%) and good sensitivity (up to 80%) (Sundaresan et al., 2016; André et al., 2019). In fact, EGFR testing was the first FDA-approved non-invasive blood-based assay and outperformed tissue biopsies in sensitivity (80% vs. 75%, respectively). Multi-gene panels (i.e., targeted sequencing) and unbiased approaches (e.g., next generation sequencing-based approaches such as WES) enable broader detection or *de novo* discovery of actionable mutations, single-nucleotide variants (SNVs), copy number alterations (CNAs), fusions, insertion or deletions (indels), and tumor mutational burden/microsatellite instability (TMB/MSI), but usually have lower sensitivity (Adalsteinsson et al., 2017; Zviran et al., 2020). Two gene-panels (Guardant360 and F1 Liquid CDx) have been prospectively validated and received FDA approval as companion diagnostics in various solid tumors. Non-mutation-based methods include fragmentomics and methylation patterns that exploit differences in fragment size, chromatin structure, epigenetic marks, histone binding and transcription factor occupancy between healthy tissue and tumor-derived DNA (Shen et al., 2018; Cristiano et al., 2019).

In breast cancer patients, ctDNA levels can predict for pCR (Rothé et al., 2019), relapse (>6 months before clinical detection) (Garcia-Murillas et al., 2015) and MRD (McDonald et al., 2019) and have been investigated as a predictive biomarker to guide targeted therapies (Turner et al., 2020b), immunotherapies and monitoring resistance, e.g., to known mechanisms such as ESR1 mutations (Schiavon et al., 2015). Unbiased technologies such as WES do not require *a priori* knowledge of genomic alterations and can uncover novel resistance mechanisms (Murtaza et al., 2013). Interestingly, genomic analysis of ctDNA can infer micrometastases signals and cancer dormancy in breast cancer patients (Shaw et al., 2012; Bettegowda et al., 2014), potentially guiding therapy to minimize both over- and under-treatment in early-stage cancers (Olsson et al., 2015). The possibility to screen for cancer in healthy persons has been explored but so far lacks clinical evidence (Merker et al., 2018).

### 3.3 Therapeutic Strategies Targeting Dormant, Micrometastatic Disease

Dormant tumor cells might play a critical role in therapy failure and have been described to be drug tolerant, i.e., phenotypically/transiently able to withstand (adjuvant) systemic therapy rather than being drug resistant, i.e., genotypically/permanently drug-resistant as demonstrated *in vitro* and *in vivo* (Sharma et al., 2010; Hangauer et al., 2017). Innovative clinical trials have been designed exploring specific targeting strategies against DTCs in breast cancer patients (NCT01545648, NCT03572387, NCT00248703, NCT03400254, NCT03032406, NCT02478125, and NCT04841148), yet no eradication strategy has been implemented into routine clinical care to date.

Three principal strategies have been envisioned to target residual dormant disease: 1) enforcing dormancy, 2) awaken dormant cells and target them with antiproliferative strategies or 3) eradicate dormant cells. A combination of all strategies might be considered to avoid escape and relapse.

#### 3.3.1 Enforcing Dormancy

The success of prolonged hormone deprivation in ER-positive breast cancer patients can likely or at least partially be attributed to preventing the outgrowth of dormant DTCs (Davies et al., 2013). Similarly, CDK4/6i inhibit cell cycle progression and induce cell cycle arrest as demonstrated in various clinical trials (Johnston et al., 2020; Loibl et al., 2021; Gnant et al., 2022). A pre-clinical study identified a small molecule agonist (C26) of NR2F1, which induces a potentially selfsustained dormancy program in cancer cell lines and *in vivo* models (Khalil et al., 2022). Reactivation of other dormancy factors, either autocrine [e.g., p38 MAPK (Aguirre-Ghiso et al., 2003), DYRK1A (Chen et al., 2013)] or paracrine/niche-related [e.g., BMP4 and 7 (Kobayashi et al., 2011; Gao et al., 2012), TGF-β2 (Bragado et al., 2013), type 3 collagen (Di Martino et al., 2022)] can maintain cancer cell dormancy in experimental models. Inhibition of integrin signaling can maintain a dormant state *in vitro* (Barkan et al., 2008) and several integrin-targeted antibodies (i.e., intetumumab, abituzumab, and volociximab) have been developed. Dormancy could further be promoted in experimental breast cancer models *via* the inhibition of lysophosphatidic acid receptor 1 (LPA1), which induces a p38^high^/ERK^low^ state (Marshall et al., 2012). Targeting various kinases, e.g., proto-oncogene tyrosine-protein kinase Src (Src) and ERK *in vitro* (Barkan et al., 2010), or urokinase plasminogen activator surface receptor (uPAR) *in vivo* (Aguirre Ghiso, 2002) can also enforce dormancy.

Homeostasis and metabolism related vulnerabilities of dormant DTCs offer additional opportunities to target these sleeper cells. A recent study demonstrated that MitoQ, a mitochondria-targeted ROS inactivator, can enforce cellular dormancy and prevent metastatic relapse in mouse models of breast cancer (Capeloa et al., 2022). This compound already passed phase 1 studies with acceptable toxicity and is slated for phase 2 trials. Based on clinical data the anti-diabetes drug metformin has been shown to reduce Ki-67 expression in breast cancer (DeCensi et al., 2014). A randomized trial (CCTG MA.32 phase 3 trial) using adjuvant metformin failed to show overall benefit regarding relapse and survival as reported at 2021 San Antonio Breast Cancer Symposium, but a positive signal could be detected for HER2-positive patients. Since the trial was not adequately powered for this subgroup, analysis follow-up investigations are required. As demonstrated in a mouse model, stress hormones (e.g., cortisol, epinephrine, norepinephrine, and serotonin) can awaken dormant tumor cells *via* the production of the pro-inflammatory proteins S100 calcium-binding protein A8 (S100A8) and S100A9 by neutrophils (Perego et al., 2020). This effect could experimentally be mitigated by beta-blocker treatment. Inflammation as a trigger to reactivate dormant cancer cells provided the rational for both the Aspirin after Breast Cancer (ABC) (NCT02927249) and ADD-ASPIRIN trials (NCT02804815). The former was recently reported at the annual American Society for Clinical Oncology (ASCO) meeting and showed no improvement in disease-free survival or overall survival for high-risk, early-stage breast cancer with 5 years daily aspirin vs. placebo. Results for the latter are pending.

Disadvantages of the dormancy-maintenance strategy are the requirement for potentially indefinite treatment (with associated cost and toxicity), not addressing tumor mass dormancy and remaining MRD with the risk of escape and relapse.

#### 3.3.2 Awakening Dormant Cells

The rationale behind this strategy is to render non-proliferating tumor cells susceptible to antiproliferative drugs including agents already currently used in the adjuvant setting, for example, by reverting drug-induced dormancy through drug holidays (Recasens and Munoz, 2019). Drug-tolerant persister cells might only acquire phenotypic (hence reversible) drug resistance, for example, be entering a dormant state, instead of genotypic (or irreversible) resistance, that can be overcome by removing the selective pressure of drug exposure (Vallette et al., 2019; Mikubo et al., 2021). Additional strategies include treatment with granulocyte colony stimulating factor (G-CSF) (Saito et al., 2010) or a small molecule checkpoint kinase 1 inhibitor GDC-0575 (Di Tullio et al., 2017) which were successfully used in preclinical studies to drive dormant cancer cells back into a cycling state. Inhibition of osteopontin (OPN) in the metastatic niche *via* anti-OPN antibodies promotes cell cycle transition and progression of dormant cells in mice (Boyerinas et al., 2013).

Most of these studies have been performed in hematological malignancies, based on similarities between hematopoietic stem cells and dormant leukemic cells. In remains unclear whether dormant breast cancer DTCs are susceptible to these strategies and whether these treatments might exhaust the healthy hematopoietic stem cell compartment. Finally, a major disadvantage of this strategy could be the awakening of aggressive or tolerant subclones that regrow metastatic lesions even in the presence of antiproliferative drugs (Sharma et al., 2010).

#### 3.3.3 Eradicating Dormant Cells

Inhibition of AMP-activated protein kinase has been shown to kill dormant breast cancer cells in ER-positive preclinical models (Hampsch et al., 2020). Interestingly, while individual targeting of Src or ERK maintains dormancy *in vitro* (Barkan et al., 2010), a combinatorial approach using a Src (AZD0530) and the mitogen-activated protein kinase1/2 (MEK1/2) targeting inhibitor (AZD6244) eradicates dormant breast cancer cells both *in vitro* and *in vivo* (El Touny et al., 2014). Epigenetic targeting *via* inhibition of lysine (K)-specific demethylase 5 by CPI-455 or targeting autophagy with inhibitors such as hydroxychloroquine (HCQ), 3-methyladenine or bafilomycin can kill dormant breast cancer cells *in vitro* and *in vivo* (Vinogradova et al., 2016; Vera-Ramirez et al., 2018). Zoledronic acid can prevent bone metastasis potentially *via* the elimination of DTCs in breast cancer patients (Banys et al., 2013).

Several preclinical studies and targeted approaches against dormant DTC have been described in other cancer types such as CNS tumors (medulloblastoma, glioblastoma), pancreatic ductal adenocarcinoma, lung and ovarian cancer. Examples include insulin-like growth factor (IGF) pathway inhibition (pancreas), antibiotic treatment such as mithramycin and oligomycin (pancreas and medulloblastoma), and immune targeting [e.g., PD-L1 chimeric antigen receptor (CAR)-NK cells plus IL-15 superagonist (N-803), reactivation of INF β ] (Recasens and Munoz, 2019). Although usually referring to a reversible phenotype, dormancy potentially comprises a spectrum of growth arrest states, including senescence. Hence, senolytic drug are an area of active investigation in adjuvant cancer therapy (Carpenter et al., 2021).

While the killing of dormant DTCs could result in the eradication of MRD, universal applicability or efficacy is not guaranteed, creating the risk of transformation or persistence of (potentially more aggressive) subclones.

## 4 Challenges and Priorities

Several important questions remain: What is the role of DTC clusters and what cell types do they associate with? Why do DTCs home to specific target organs and what are the distributing factors? What diagnostic tools and biomarkers are best suitable to detect and target dormant cancer? Can we target dormant disease in the clinic and detect or ensure successful cancer control? Can we apply this strategy in the metastatic setting? How can this be implemented in clinical trial design?

Based on the current literature review presented here, we believe that to realize the goal of curative cancer care, both further biological understanding of micrometastatic dormant disease as well as clinical-translational efforts are needed. Once macrometastatic disease arises, treatment is very challenging and rarely curative. Understanding the nature of DTCs and DTC clusters will likely have a similar fundamental impact as the dissection of CTCs and CTC cluster biology. Hence, the field of tumor and cancer cell dormancy would greatly benefit from advanced models to reliably capture biology and vulnerabilities. Such knowledge could lead to a paradigm shift in adjuvant therapy, moving us closer to preventing lethal metastatic relapse by targeting not only proliferating cells but also dormant DTCs and micrometastasis.

To design efficient therapies, adequate predictive biomarkers are of paramount importance and novel diagnostic tools are needed. Current personalized clinical decision-making in the oncology field focuses on molecular profiling of macroscopic tumors, either primary or metastatic, to identify actionable targets. These approaches are problematic: firstly, a primary tumor may be (genotypically and phenotypically) very different from macrometastatic lesions (e.g., many ER-positive switch to ER-negative breast cancer in the metastatic setting), which might be different from DTCs and micrometastasis (e.g., proliferating vs. quiescent). Secondly, tissue biopsies can become extremely challenging when not readily accessible. Thirdly, sampling bias due to tumor plasticity and heterogeneity is likely to occur.

We believe that liquid biopsies and in particular CTCs can be leveraged to improve upon these shortcomings. Because every organ has access to the circulation, CTCs and ctDNA analysis may enable, from a theoretical standpoint, the full coverage of (micro-) metastatic cancer heterogeneity. Major challenges towards the clinical implementation of CTCs and ctDNA include the physiological scarcity compared to other blood components, resulting in ultra-low harvesting yields, short half-life in circulation [minutes for CTCs (Aceto et al., 2014) and <2 h for ctDNA (Yu et al., 2013)], occult anatomical tumor sites, and lack of universal pre-, intra- and post-analytical standards. Regardless of the CTC capture technology used, biases are inevitable, such as selecting high-antigen expressing cells when using *a priori* marker profiles or selecting larger CTCs in the case of size-based methods (Castro-Giner and Aceto, 2020). ctDNA isolation is considered less technically challenging but cannot offer the same range of biological readouts or functional studies. Multiple protocols exist for ctDNA isolation that lack broadly implemented standardization for clinical application and reliable data on comparability (Leest et al., 2020). The scarcity of CTCs and ctDNA can results in false negative testing even in patients with advanced cancers due to low tumor burden, anatomically occluded sites (e.g., CNS), low proliferation and apoptosis, poor vascularization and limited detection of potentially relevant subclones and subclonal mutations (Razavi et al., 2019; Peneder et al., 2021). Because ctDNA predominantly originates from dying cells (Diaz and Bardelli, 2014), and not all mutations are expressed, information about minor resistant sub clones that could ultimately drive disease progression can be missed. Other confounding factors include new primary tumors, clonal hematopoiesis of undetermined significance (CHIP) and somatic mutation “field defects” that accumulate in healthy tissue with age (Martincorena et al., 2018; Razavi et al., 2019). Finally, no head-to-head assessment exists comparing ctDNA and CTCs utility for detecting and monitoring MRD or dormant disease.

### 4.1 Clinical Trial Design

After the boon years, recent development shows disappointing returns from late phase clinical trials using conservative designs: smaller improvements in outcomes, increased toxicities, negative findings and longer trial durations due to low event rates (Piccart-Gebhart et al., 2016; von Minckwitz et al., 2017). In 2021, success in improved treatment for oncological patients was mostly represented by surrogate endpoints (DFS), progression-free survival, and sometimes even pathologic complete response). These measures do not always translate into OS or improved quality of life and come at a high cost (Piccart, 2013).

**FIGURE 3:**
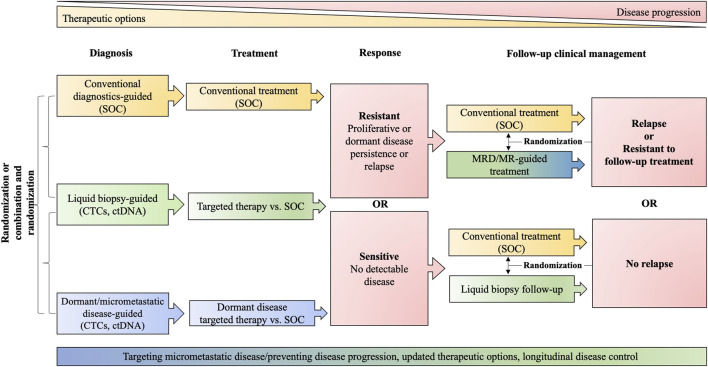
Clinical trial design. The schematic proposes the randomization and comparison of standard of care (SOC) approaches (yellow) with liquid biopsies generally (green) and approaches specifically addressing dormancy (blue). The red color indicates stages in clinical management that trigger further decision making. MRD, minimal residual disease, including dormant disease; MR, molecular relapse; ctDNA, circulating tumor DNA; CTCs, circulating tumor cells; SOC includes diagnostic modalities: tissue biopsies, imaging, conventional biomarker; and guideline-conform treatment.

We believe that innovative trial design is needed, aimed at reducing toxicities and cost while improving long-term survivorship with strong emphasis on predictive biomarker discovery through translational research, incorporation of liquid biopsies for non- or minimally invasive, continuous and dynamic sampling (and potential initiation of “rescue” therapies) and rational targeting of micrometastatic disease [e.g., c-TRAK-TN trial for TNBC (NCT03145961)] (Figure 3). Dormancy makers [e.g., NR2F1 (Borgen et al., 2018)] ought to be included in trial design and sampling of different compartment (e.g., BM) should be evaluated. The exploration and validation of dormancy markers could be achieved in a relatively short time by investigating patients currently under standard of care treatment with CDK4/6i or anti-hormonal therapy. A current trial (NCT02732171) is attempting to screen breast cancer patients for the presence of BM DTCs and potential inclusion of positive patients into other dormancy targeting trials mentioned above. Another trial pursues a somewhat riskier strategy by mobilizing DTCs from BM using a small molecule inhibitor of E-selectin and CXCR4 (GMI/1359) in combination with CTC enrichment (NCT04197999). Trials combining antiproliferative and dormancy-targeted approaches are also underway [e.g., combining mammalian target of rapamycin (mTOR) inhibitors with HCQ] (NCT03032406). Since the integration of intensive translational research and proper evaluation of patient eligibility is associated with scientific and logistical hazards, industry-academia partnerships should aim to control cost and enable rigorous, translationally oriented analysis.

## 5 Outlook

Early, minimally invasive detection and subsequent targeting of DTCs *via* liquid biopsies can be considered an essential prerequisite to improve outcomes in breast cancer patients. We and others have already leveraged knowledge gained through mechanistic investigations of CTCs and CTC clusters for the design of clinical trials to specifically target metastasis. We anticipate that the identification of clinically relevant and therapeutically actionable biomarkers for dormant micrometastasis prone to relapse will provide further, highly innovative solutions to an unmet need in clinical practice, i.e., the identification of the best possible treatment strategy for patients with dormant cancer cells before the emergence of aggressive, macrometastatic cancers that are resistant to standard-of-care treatment. We believe that a multiple-analyte approach combining the virtues of different liquid biopsies can overcome current limitations and will provide a powerful tool for future prognostic and predictive biomarkers, enabling a more complete representation and targeting of the heterogeneity of DTCs to unlock the full potential of comprehensive adjuvant cancer care. Adjuvant therapy not only targeting dividing cells but also dormant DTCs and their capability to spawn relapse arguably represents our best opportunity to prevent lethal macrometastasis and improve outcomes.
